# Assessing care deficits in Ireland's international protection accommodation system: Lessons learned in COVID-19 and beyond

**DOI:** 10.1016/j.jmh.2024.100255

**Published:** 2024-07-25

**Authors:** Felicity Daly, Jacqui O'Riordan

**Affiliations:** a Assistant Professor Global Health (2023 – Present), Trinity Centre for Global Health, Trinity College Dublin, the University of Dublin, 7-9 Leinster Street South, Dublin D02 K104, Ireland; b Postdoctoral Researcher (2020-2033), Institute for Social Science in the 21st Century (ISS21), University College Cork, Top Floor Carrigbawn Building, Donovan Road, Cork T12 YE30, Ireland; c Lecturer (Retired), School of Applied Social Studies, University College Cork, Ground Floor Carrigbawn Building, Donovan Road, Cork T12 YE30, Ireland

**Keywords:** COVID-19, Care deficits, Structural determinants of health, Asylum seekers, Ireland

## Abstract

Responding to the need for qualitative research that reveals the lived reality of how forced migrants endured the COVID-19 pandemic this paper presents findings from eleven interviews with asylum seekers residing in Ireland's Direct Provision (DP) accommodation system that detail care deficits before, during and after COVID-19 along with analysis of how care is discussed within Irish policy documents concerned with the health and wellbeing of asylum seekers. The research contributes personal testimony and documentary evidence of the inability of DP to properly adapt to the pandemic and its failure to protect the health and wellbeing of asylum seekers given pre-existing care deficits. The paper argues that an ethic of care practiced for and with asylum seekers must ensure they are not re-traumatised, and their health disparities are not exacerbated during public health crises and beyond. The findings are relevant to efforts to reform how international protection responsibilities are enacted in Ireland and other destinations of forced migrants, including EU member states.

## 1 Introduction

COVID-19 incidence was higher amongst forced migrants (Hintermeier et al., 2021) and pandemic compounded the vulnerability of refugees and asylum seekers, worsened health and wellbeing, deepened economic insecurity and demonstrated the need for decent accommodation (Guadagno, 2020; Bozorgmehr et al., 2020). COVID-19 lockdowns intensified poor mental health amongst vulnerable groups of forced migrants, whether by cessation of integration activities increasing the sense of disillusion and uncertainty (Da Mosto et al., 2021) or severe movement restrictions, causing symptoms of depression and stress (Saw et al., 2021).

Forced migrants often live in precarious, unsanitary and over-crowded conditions considered as violating the UN Refugee Convention (Smith, 2004). These settings present challenges to implementing measures to prevent the spread of SARS-CoV-2 such as quarantine, self-isolation, social distancing, enhanced hygiene and other mitigation efforts (Kluge et al., 2020; Raju and Ayeb-Karlsson, 2020; Hintermeier et al., 2021). Communication that does not translate or tailor appropriate and easily understood health guidance contributes to the exclusion of forced migrants from effective COVID-19 mitigation (Maldonado et al., 2020). Factors which exacerbate pre-existing barriers to health care and intensify the risk of exposure to COVID-19 are rooted in the bureaucratic operation of international protection and deemed a form of structural violence against refugees and asylum seekers (Da Mosto et al., 2021). European Centres for Disease Prevention and Control recommendations to avert outbreaks of COVID-19 in migrant and refugee centres included: physical distancing; hand and respiratory hygiene; free and equitable prevention, testing, treatment, care and communications adapted to meet the different language, cultural and literacy needs of a heterogenous population (ECDC, 2020). The UN High Commissioner for Refugees, World Health Organization, International Organization for Migration and Office of the High Commissioner for Human Rights insisted that protection of the human rights and health of vulnerable migrants be factored into inclusive COVID-19 responses (UNCHR, 2020 ).

This paper analyses how threats to the health and wellbeing of asylum seekers are represented and understood within Irish policy regarding intercultural health care provision, the response to COVID-19 and Ireland's international protection system. Having found no personal testimony from asylum seekers in the policy documents analysed, the paper presents testimony from asylum seekers who participated in a research project that considered their care experiences.

The paper emerges from a research project exploring care experiences of marginalised groups in Irish society considering the COVID-19 pandemic and beyond. The project's focus on asylum seekers is grounded in an understanding that the State holds ‘a public and care responsibility in developing supports for vulnerable populations, and in being politically accountable for its national and international care responsibilities’ (O'Riordan, 2020: 4). This paper seeks to contribute to the expressed need for ‘qualitative studies…instrumental to provide insights into realities of migrant and forcibly displaced population groups during the pandemic’ (Hintermeier, 2021: 12).

The research is conceptually framed by ethics of care which recognise the centrality of care to human life and wellbeing (Tronto, 1993) and assert care as the fundamental moral value to be upheld along with the value of justice (Held, 1995, 2006). Care ethics has been reasserted as a crucial perspective to interrogate inequities of care experienced by groups in society during the COVID-19 pandemic (Chatzidakis et al., 2020; Fine and Tronto, 2020). The policy analysis approach is informed by Sevenhuijsen's (2004) Trace method which identifies discourses within policy documents that shape how care is constructed, problematized and/or silenced. Care concerns revealed in policy documents analysis guided empirical research with international protection applicants in Ireland to explore their experiences of care before, during and after the pandemic.

This paper begins by outlining the context for Irish policy documents and then discusses methods for policy analysis and empirical research. The results present policy discourse supplemented and challenged by findings from interviews with eleven asylum seekers. The discussion utilises ethics of care concepts to deepen understanding of how the precarity of asylum seekers’ health and wellbeing can inform efforts to reform how international protection responsibilities are enacted in Ireland and other states, including EU members.

## 2 Background

Ireland responded to greater numbers international protection applicants arriving from 2000 by introducing an ‘emergency response’ to accommodate them while they wait for the Department of Justice to decide on their application. This system, known as Direct Provision and Dispersal (DP), is managed and overseen by the International Protection Accommodation Services (IPAS) to allocate shared rooms in communal spaces, in centres across the country. As of July 2020, there were 8812 individuals living in DP centres (38.1 % families and 61.9 % single individuals of which 63.7 % were male and 36.3 % female comprised of the following age groups: 17.3 % age 0–13; 3.0 % age 14–17; 43.2 % age 18–34; 35.9 % age 35–64 0,7 % age 65 and over (Government of Ireland, 2020: 31).

A few DP centres are State owned and operated but the majority operate in privately owned hotels, tourist lodges and other structures. Generally, people reside in DP for several years while they wait for a decision to be made given a backlog of applications. This period of waiting is characterised as generating feelings of uncertainty, aimlessness and isolation and has been described as ‘living liminality’ (O'Reilly, 2018; Isaloo, 2020). Asylum seekers are entitled to a minimal allowance along with room and catered meals, access to free health care and the ability to work after six months.

A growing corpus of evidence reveals that living in DP exacerbates mental distress with higher levels of self-reported PTSD, depression and anxiety associated with the length of time spent in DP (Toar et al., 2009). Mental distress experienced while waiting for a decision on an application identifies precursors of suicidal behaviour (Murphy et al., 2021). Additionally, there are concerns about access to health care for children of non-EU migrants. There is evidence of lower utilization of general practitioner services and relatively lower attendances at Emergency Departments and hospital nights for these children relative to children born to EU citizens (Mohan, 2021).

While some of these health disparities have been highlighted in government policies ‘these have not proved sufficient solutions’ (Barlow et al., 2022: 8). The emergence of COVID-19 amplified concerns about the lack of safeguarding of the health and wellbeing of residents in DP (Gusciute, 2020; Irish Refugee Council, 2020b; Murphy, 2021; Isaloo, 2021). While understanding in Ireland about social determinants of migrant health is expanding, knowledge around addressing structural barriers to improve health outcomes ‘need attention to expand the evidence base’ (Villarroel et al., 2019: 9).

## 3 Methods

This study is part of the CareVisions project which sought to explore care relations in Ireland following the COVID-19 pandemic. Two qualitative methods were utilised in this study; (1) policy analysis of how care was discussed within Irish policy processes concerned with health and wellbeing of asylum seekers and; (2) semi-structured interviews with asylum seekers which explored ways COVID 19 impacted, altered or disrupted their care; and how, if at all, has COVID-19 led to a rethinking of ways in which care practices and relations might be reconfigured in Ireland's international protection system.

### 3.1 Policy analysis

The paper presents results of a Trace analysis of documents concerned with care standards within Ireland's international protection system and the health and wellbeing of asylum seekers, including COVID-19 risks. The Trace methodology (Sevenhuijsen, 2004) is a multi-step process to determine ‘the context in which the text was produced; definitions of care utilised…the perceived role of the state vis-à-vis care; and what ‘leading values’ are at work in the text’ (Sevenhuijsen, 2004: 4).The first author began the Trace process by analysing documents produced for the Houses of the Oireachtas (Irish Parliament) Special Committee on COVID-19 Response, a short-term committee established in May 2020 with a mandate to ‘consider and take evidence on the State's response to the COVID-19 pandemic’ (Houses of the Oireachtas, 2020: 109). The Special Committee on Covid-19 Response (SC) was broadly concerned with ‘the huge challenges posed to public health, both in treating COVID-19 and maintaining continuity of care for all other health conditions, other essential State services and the economy’ (Houses of the Oireachtas, 2020: 8). Committee members invited submissions of evidence and held a limited number of hearings with Government officials and other relevant stakeholders.

The SC's work provides insight into the ways COVID-19 impacted, altered or disrupted the care experienced by a range of vulnerable populations in Ireland and with what effects (Daly and Edwards, 2022). This paper focuses on the SC's investigation of the impact of rapid spread of COVID-19 in congregated settings which included descriptions of the vulnerability of asylum seekers within DP centres. To provide greater detail on these vulnerabilities, a set of related documents were analysed including: submissions and statements made to the SC by the Irish Human Rights and Equality Commission and the Irish Refugee Council; analysis of video and print transcript of the SC's session on Direct Provision; and guidance issued by the Department of Justice and Ireland's Health Service Executive for the protection of people living in DP from SARS-CoV-2 infection. Review of these sources deepened understanding and allowed for consideration of perspectives of community-based organisations representing forced migrants.

To contextualise the SC's care discourse, an additional phase of analysis was undertaken by both authors to identify relevant content in: the *Report of the Advisory Group on the Provision of Support including Accommodation to Persons in the International Protection Process* (Government of Ireland, 2020); *A White Paper to End Direct Provision and to Establish a New International Protection Support Service* (DCEDIY, 2021) and the *Health Service Executive Second National Intercultural Health Strategy 2018–2023*. The Advisory Group report was selected for analysis as their investigation was undertaken during the pandemic and revealed care failures and disparate health impacts. The White Paper was selected for analysis because it identifies health and wellbeing concerns were revealed and intensified in the COVID-19 pandemic, and outlines an enhanced model of community health services for asylum seekers. The Intercultural Health Strategy was selected as the Health Service Executive (HSE) maintains oversight for community health services and outlined strategic actions to address asylum seekers’ health disparities.

### 3.2 Empirical research

Because no personal testimony from asylum seekers was identified in the documents analysed, qualitative interviews aimed to provide an opportunity for their voices to supplement and challenge public discourse. Framing research questions for this empirical research explore in what ways COVID 19 impacted, altered or disrupted their care networks, and with what effects; and how, if at all, COVID-19 led to a rethinking of ways in which care practices and relations might be reconfigured for asylum seekers. Inclusion criteria for this purposive sample was people over age 18 who have applied for international protection in Ireland, the majority still resident in DP at time of interview. Ethical approval for the research protocol was granted from University College Cork Social Research Ethics Committee.

Interviews pursued three strands of enquiry, the first informed by findings of the policy analysis were specifically designed to explore the personal impacts of conditions within DP during COVID-19. This context framed semi-structured interviews conducted online between April and June 2022 with four individuals: two men and two women, all from sub-Saharan African countries. These interviews sought to determine what participants perceived as the COVID-19 specific challenges of living in DP during COVID-19 lockdown and to examine the circumstances of being transferred between DP centres. The Movement for Asylum Seekers (MASI) assisted with identification of participants in this part of the sample given the organisation's campaigning to address COVID-19 risks facing asylum seekers.

Two other strands of enquiry explored care deficits and risks to health and wellbeing experienced by DP residents through seven semi-structured interviews conducted by both authors. This includes findings from online interviews conducted between June and September 2022 with a sample recruited with support from Cork Migrant Centre of five women, also all from sub-Saharan Africa who described how living conditions within DP presented health risks and compromised their ability to work in the health and care sector during the COVID-19 pandemic. It also includes findings from two semi-structed interviews, conducted in person by the first author outside a DP centre. Clonakilty Friends of Asylum Seekers facilitated introductions to DP residents. This part of the sample comprises one woman from sub–Saharan Africa recently arrived in DP with her infant at the time of interview and one woman from South Asia who at the time of interview had been residing in DP with her husband and two young children for seven years. These interviews reveal care standards within DP before and after COVID-19. Anonymised findings from a sample of eleven individuals add to policy analysis and provide the lived reality of care deficits that exacerbate health and wellbeing challenges amongst residents of DP.

## 4 Policy analysis and empirical findings

Four themes from policy analysis and interviews with asylum seekers are presented here: care deficits in the international protection system; COVID-19 risks in DP conditions; health outcomes of transferring asylum seekers between DP centres; and how COVID-19 informed international protection policy and practice.

### 4.1 Care deficits in the international protection system

The HSE acknowledges that Ireland's health system has challenges in ‘provision of health and other support services to fully meet the diverse social and healthcare needs of migrants, particularly vulnerable migrants and asylum seekers’ (HSE, 2017: 47). The HSE Intercultural Health Strategy (2018–2023) identifies vulnerabilities of asylum seekers relate to ‘displacement, loss of family members, and physical and emotional trauma, war and sexual violence in their home countries…(they) experience significant health and care needs’ (ibid: 51). Yet there is little evidence of community health services’ capacity to meet these health and care needs. Moreover, tertiary care fora range of conditions and disabilities are mainly located in the capital city, Dublin. When IPAS allocates asylum seekers space in DP centres across all counties it does not seem to take need for specialised care into account. One participant discussed how this leads to care deficits amongst residents in her DP centre. She conveyed,

‘Some people have specific reasons [they] don't want to go to a certain place because of a medical condition…For example, there was a family that had two kids with diabetes…they were brought to this place…the public transport, it's very big problem…If you want to go to services outside, it's not easy… they have to go and attend a diabetes clinic most of the time and they have to travel from here to almost…Dublin. Because there's no diabetic clinic here’ (Participant - D).

Another participant raising a child that was diagnosed with autism spectrum disorder spoke about care deficits she has experienced at multiple levels throughout seven years that she, her husband and two young children have lived in one room in DP. She said,

‘these kind of children they don't get any support worker. But in here I needed at that time, because the one room and I couldn't manage him. When I was pregnant, they [a community-based organization supporting people with intellectual disabilities] provide me one support worker. But that was very difficult…In this Direct Provision centre, from them, honestly speaking, I didn't get anything’ (Participant - B).

Lack of capacity to effectively respond to the care needs of a family with a child with autism amplified after her second baby arrived. She recalled,

‘Everyone has the one room there is no second room here. But they can't do anything because it comes from upper level…IPAS…I couldn't get any help. I cried like still at least give me, for three months at least, give me one room…they didn't give me any room. So, after six months I stopped breastfeed my daughter.… I can't because when I woke up to breastfeed my daughter my son was crying, he get upset, he screamed. So, then I stopped after six months. That was very sad thing for me’ (Participant - B).

While she was able to adhere to the recommendation for exclusive breastfeeding for the first six months of life, the strain on two adults raising two small children, one with special needs, confined for years to one congested hotel room cannot be underestimated.

### 4.2 COVID-19 risks in DP conditions

The SC identified DP as a particularly problematic congregated setting with inherent weaknesses that undermine the ability to implement communicable disease prevention. By the time the SC concluded in September 2020, 14 outbreaks in DP centres had occurred with 175 COVID-19 cases (Houses of the Oireachtas, 2020). This represents 1.9 % of people resident in DP and 0.4 % of COVID-19 cases in Ireland at that time (Government of Ireland, 2020; Government of Ireland, 2024). The SC documented that the spread of COVID-19 was facilitated in DP centres as residents ‘were not able to self-isolate when they fell victim to the virus or when they displayed symptoms prior to diagnosis’ (Houses of the Oireachtas, 2020: 17). The SC requested that the Oireachtas Joint Committee on Children, Disability, Equality and Integration ‘review the practice of accommodating people seeking international protection in direct provision centres, hotels and B&Bs with a particular focus on the need to provide appropriate living accommodation for individuals and family units.…put a protocol in place to ensure the that appropriately qualified staff are recruited, and Garda [police] vetted particularly in the context of dealing with vulnerable adults and children… ensure that all residents in congregated settings are tested for Covid-19 routinely…ensure that staff working in direct provision services receive appropriate training, including in infection control measures’ (Houses of the Oireachtas, 2020: 36).

The Irish Refugee Council's submission to the SC raised alarm about overcrowded conditions in DP and detailed that, contrary to the State's instructions, residents continued to have to share intimate space such as bathrooms, dining areas, communal living spaces and laundries (Irish Refugee Council, 2020a). Testimony from MASI also articulated its consistent concerns about the ‘warehousing of asylum seekers.…in inhumane conditions with overcrowding in crammed conditions in for-profit direct provision centres.…people have been stripped of their fundamental human right to privacy and the dignity that comes with it’ (Houses of the Oireachtas, 2020: 37).

Nine participants unanimously confirmed that measures to protect people living in DP did not conform to the COVID-19 mitigation guidance issued by the Department of Justice and the Health Service Executive (HSE) (Department of Justice and Equality, 2020). They revealed a sense of increased vulnerability during the pandemic andand conveyed that staff in DP centres were unable to effectively implement public health guidance within overcrowded DP centres. A participant reflected on how the onset of the pandemic strained interactions with DP centre staff stating,

‘we obviously got the information.…that pandemic is spreading and it's coming to Ireland and there's been a few cases.…the attitude and the response from the management changed towards.…asylum seekers, suddenly.…they didn't even want to come close.…and we understand social distancing was one of the measures that we had to take. But at some point, even communicating was a problem.… they will scream at you.…And it was actually affecting us because we felt like we were in a vulnerable state’ (Participant - G).

Five participants working in the health and social care sector during COVID described how prevention measures were not adequately applied within congested, shared spaces and facilities inherent in DP which compromised their ability to work. A participant shared ‘proper procedures according to health weren't put into place, especially now that COVID had already surfaced. Care was not taken into consideration’ (Participant - L). Furthermore, they recalled how measures to mitigate COVID-19 fell short, stating ‘the outbreak began.…even if you wanted to be tested or to take precautionary measures yourself, you would not have that option’ (Participant - L). This account was supported by another participant who said, ‘we didn't have PPE.…there was no supply, and we were still congested - four in a room’ (Participant - G). Yet another participant spoke about how it was impossible for DP residents to self-isolate following a positive COVID test and e that a man living in her DP centre with his wife and three children had tested positive. She recalled the family was ‘staying in one bedroom. But he was the only one who was positive. The rest were negative, but they had to isolate. How are they going to isolate in the same room? So, they had to stay without isolation’ (Participant - D).

Participants conveyed that communications did not take account of linguistic diversity. HSE COVID-19 guidance posted in DP centres was not translated from the official languages, English and Irish (Gaeilge), into any of the languages understood by population groups resident in DP. A participant stated,

‘there was no adequate information at that time.…Some of the people that were there they couldn't speak English.…they don't even know or understand how the COVID spreads.…the HSE put a leaflet there, but nobody will read…they should talk to them; ‘please do this’. Like someone telling people in their language’ (Participant - P).

One participant stressed that she tried to negotiate with DP staff so that they didn't have to touch so many surfaces and asked staff if corridor doors could be kept open. She was told that those are fire doors which cannot remain open, yet such a door remained open in the reception area where staff worked. This participant lamented ‘for the staff then, they're reducing their exposure… but refusing to do a similar thing for residents - That's a two-tier system, basically’ (Participant - V).

Another participant who resided in a DP centre for two years from March 2020, said ‘I'm gonna be frank. We did not see any strong measures being undertaken like testing your temperature when you're coming in.… the situation in the in the asylum DPs are not the same’ (Participant - G). During outbreaks that occurred in that location this participant recalled ‘you are locked in for 10 days, which is more like imprisonment. And so, we don't want someone to bring the virus to us and then we'll get locked in for 10 days’ (Participant - G). Participants found prolonged periods of time in isolation during COVID-19 outbreaks within DP centres extremely difficult. One recalled ‘when they discovered there were two COVID cases.…then they locked us down. We were inside, we were isolated for like, 30 straight days…I kept my son in the room for 60 days’ (Participant - P). Another participant, living in DP with a young child, revealed that they had endured traumatic life experiences before leaving their country of origin and seeking asylum in Ireland. Yet, she asserted that lockdown ‘was the most horrible time of our lives. We had 14 days inside.…because outbreak, numbers kept on growing, they say plus another 14, inside. It was just another traumatic experience’ (Participant - L).

### 4.3 Health outcomes of transferring asylum seekers between DP centres

A measure taken by IPAS, ostensibly to avert COVID-19 outbreaks in overcrowded DP centres, was to transfer groups asylum seekers to new accommodation., The Department of Justice testimony to the SC indicated that ‘four centres were created to cater for those required to self-isolate while 600 people were moved to other locations to reduce density in centres’ (Houses of the Oireachtas, 2020: 36). MASI testified that it became ‘increasingly concerned when measures to combat the spread of Covid-19 were announced.…people were moved to commercial hotels or new centres rather than to single rooms or family units’ (ibid: 37).

SC evidence highlights conditions in one of these new DP centres regarded as emblematic of inadequate protection of the health and wellbeing of asylum seekers wherein A large group of asylum seekers were moved at very short notice from several different DP centres in Dublin to Cahersiveen, County Kerry, a distance of over 350 km. The SC detailed a range of care deficits in this case: a lack of consultation with asylum seekers being relocated; the rushed opening of a tourist lodge as a DP centre for the first time; problems with water and heat, and DP staff who had not completed a standard police background checks necessary for work with vulnerable populations. These factors led to a rapid outbreak of COVID-19 once asylum seekers began residing at this centre, representing close to a quarter of residents (Irish Human Rights and Equality Commission, 2020). This case galvanised the SC's attention on IPAS’ mismanagement of the COVID-19 crisis; one member of the SC regarded it as ‘at the very least … a grave oversight and at worst an unequivocal dereliction of duty of care to all concerned…it is utterly shambolic and unacceptable’ (Foley, 2020).

A key aim of this study sought to surface personal testimony about the impacts on asylum seekers who were relocated to the new DP centre in Cahersiveen. Interviews further revealed factors fuelling SARS-CoV-2 transmission during the transfer included: not testing residents before boarding transport between DP centres; lack of masks or other basic PPE available during travel or afterwards; and a lack of cleaning and disinfection of common areas at the new accommodation centre once residents arrived. While transfers were intended to mitigate overcrowding, in reality they exacerbated risks of SARS-CoV-2 infection. . A participant confirmed the lack of consultation with DP residents before being moved on short notice remembering, ‘we got a letter from HSE that, quickly, we had to be transferred. You know, as part of the measures to.…mitigate the problem’ (Participant - G). Another participant remembered his reaction to being advised of the transfer by DP staff conveying IPAS’ instructions to international protection applicants and the outcome of this decision. He stated ‘I saw the (DP) manager. They came to give me a transfer letter to be transferred to Kerry, Cahersiveen. Oh my God, that was even worse’ (Participant - P). Another participant recalling how this decision was communicated and the failure to implement pandemic guidance precautions during transport between Dublin and Cahersiveen, a journey of over five hours. She said,

‘We were just given a letter ‘on such and such a date you are going to such and such a place’. That is how we were transferred.…proper care, proper channels were not followed before transferring us from.…point A to point B.… we had no masks, no [hand sanitizer] nothing’ (Participant - L).

Deleterious conditions at the new DP centre in Cahersiveen came to the public's attention through social media, amplified by MASI and other migrants’ rights organisations, picked up in national press. This case became one of the most notorious examples of the failure of duty of care within DP during the pandemic (Irish Refugee Council, 2020b; Murphy, 2021). On arrival asylum seekers found that the precautions fell far short of the recommended HSE guidance. A participant stated that ‘precautions were put into place when.…it was too late. Because precautions were supposed to be in place prior to the arrival.…As well as training and information.…it was supposed to be done prior to the arrival’ (Participant - L).

Commenting on the resulting COVID-19 outbreak a participant remembered ‘I experienced the outbreak when I was moved from Dublin to Cahersiveen. That place [shakes head] the conditions were not good.…COVID had spread like a, like wildfire.…it was so difficult.…The place was.…not fit for purpose’ (Participant - L). Following the rise of COVID cases at the Cahersiveen DP centre residents attempted to a communicate with IPAS as one participant reported,

‘People were testing positive all the time.!…We were so scared. People were crying.…It was the worst thing EVER.!…we started having people speak to IPAS.…to say ‘if you guys cannot fix this living arrangement.…we're still overcrowded.…you need to move us out.… And all IPAS had to say was.…‘unfortunately, we cannot move anyone right now because of the pandemic’. And that's it’ (Participant - T).

These findings suggest that IPAS’ attempt to address COVID-19 risks inherent in the congregated setting of DP did not mitigate outbreaks when groups of asylum seekers were transferred to unprepared, unhygienic and congested spaces. IPAS was found to be unable to arrange suitable alternatives for safe accommodation and communication between IPAS and asylum seekers did not convey an ethic of care. These decisions had identifiable health impacts, exacerbated transmission of SARS-CoV-2 and re-traumatised of asylum seekers already experiencing pre-migration and post-migration stress compounded by the mental health impacts of the COVID-19 pandemic.

### 4.4 How COVID-19 informed international protection policy and practice

Policy analysis provides evidence of the intentions to learn lessons from COVID-19 to safeguard the health and wellbeing of people seeking international protection in Ireland. One of the key recommendations from the SC instructed the State to ‘phase out support for facilities where residents do not have adequate self-isolation facilities and it should accelerate the capital works in all publicly owned facilities to ensure that all residents can live in self-contained units’ (Houses of the Oireachtas, 2020: 17). Representatives of the Department of Justice, were forthright in acknowledging DP's ‘weaknesses’ (Houses of the Oireachtas, 2020: 37). When called before the SC, the Department of Justice indicated that a report, including recommendations to IPAS, was forthcoming from *the Advisory Group on the Provision of Support Including Accommodation to Persons in the International Protection Process* (Advisory Group). The Advisory Group's investigation commenced before the onset of the pandemic and continued during the initial waves in 2020. Their recommendations were released immediately before the SC concluded in 2020 and were cited as evidence which should be utilised to reform Ireland's international protection system.

The Advisory Group found that COVID-19 had a ‘direct impact on direct provision centres, underlining their unsuitability as long-term accommodation for large groups of people’ (Government of Ireland, 2020: 5). Their report states that ‘a system which places applicants for long periods in segregated, congregated accommodation with little privacy or scope for normal family life is not fit for purpose. The arrival of COVID-19 in Ireland highlighted the risks of congregated living in direct provision and emergency centres and has added emphasis to the need to end the current system’ (Government of Ireland, 2020: 7). Escalating their concerns about the extant health risks of living in DP, the Advisory Group asserted that the pandemic ‘has shown that these congregated settings are ill equipped to deal with outbreaks of infectious diseases.…recent COVID-19 outbreaks in several direct provision centres has highlighted the unsuitability of close living in congregated settings’ (Government of Ireland, 2020: 20–21). They recommended that sufficient space for quarantine facilities be factored into plans to reform international protection accommodation as ‘the need for and lack of quarantine facilities has been highlighted in the recent COVID-19 outbreaks in DP’ (Government of Ireland, 2020: 63). Furthermore, the Advisory Group recognised that the DP system generated structural determinants of asylum seekers mental health and that there is lack of trauma informed care available to them. They recommended that international protection pplicants suffering from trauma ‘should have access to appropriate expert care’ (Government of Ireland, 2020: 133).

A new Programme for Government issued in October 2020 included a commitment to end the DP system and replace it with a new approach. A framework to enact this commitment was put forward in a *White Paper to End Direct Provision and to Establish a New International Protection System* in February 2021. The White Paper judged the DP system as ‘expensive, inefficient, and ill-equipped to respond to shifting trends in international migration.…it failed to respect the dignity and human rights of individuals’ (DCEDIY, 2021: 12). The White Paper acknowledges that ‘one of the repeated criticisms of DP is that problems were allowed to escalate and become chronic before being addressed. The new model must ensure that it identifies and addresses issues affecting applicants’ wellbeing at the earliest possible stage’ (ibid: 71). Crucially, the White Paper outlined an ‘enhanced model of community health care’ which recognises the importance of safeguarding the physical health and mental wellbeing of asylum seekers and urges that tertiary care and specialised support be made available to asylum seekers. This framework recommends that ‘targeted mental health promotion and prevention actions should recognise the distinct needs of applicants for International Protection’ (ibid: 58).

The risks inherent to congregated accommodation which made implementation of public health precautions challenging or impossible led to a SC recommendation that international protection applicants should live in self-contained units. This was echoed by the Advisory Group's call for ‘own door’ accommodation. However, the White Paper's conception of ‘own door’ accommodation only outlines separate units for families, single asylum seekers would have their own room in shared accommodation. This seems to have diluted recommendations which were based on the lessons learned in the pandemic and would not be an adequate solution to the structural health determinants of the majority of individuals who arrive in the international protection system without another family member.

All policy documents analysed urged significant changes to the treatment of asylum seekers, a call echoed by the voices of our research participants. A concluding question in all interviews asked participants to recommend changes that they believe could improve the standard of care they receive within Ireland's international protection system. One participant recommended that a framework of care be put in place to ensure that there are ‘people on ground to take care of them’ (Participant - P). Another participant indicated that management and staff at DP centres should be ‘educated more on handling.…people [who] come from different traumatic backgrounds’ (Participant - L). One participant's suggestion on how to improve the standard of care they experience in DP is simply ‘be nicer, be more caring.…Care for us as you'd care for your relative. We're not here to do any bad. We just want to be safe’ (Participant - T).

The government demonstrated some accountability for the crises in DP during the pandemic. A participant recalled that the Minister for Justice and Equality wrote to all residents of the DP centre in Cahersiveen, County Kerry yet his apology did little to assuage the long-term impacts of living in DP or to provide significant alternatives to this accommodation. This participant had endured the outbreak in Cahersiveen and was eventually moved to another DP centre. She lamented ‘I'm still under the same situation.…nothing different, still struggling the same struggles.…the Minister had previously written a letter to apologize to us.…So what exactly was being apologized to me because I still find myself in the same place?’ (Participant - L).

While participants reveal slow progress on improving the standard of care for international protection applicants, a participant who first arrived in DP in May 2022, provided a positive assessment. She shared,

‘Since I've been here in Direct Provision…one thing I'm especially grateful for the healthcare system. I wouldn't know if it was supposed to be better than this because I have not been in this system for too long. But coming from where I was, I think it's very good…We have a medical card, and we can get some drugs for free’ (Participant – N).

Nevertheless, she identified that differential assessments of the vulnerability to viral outbreaks continues in stressing that asylum seekers should be able to access flu vaccinations offered by the HSE for the elderly, health care workers and a select number of other groups but not DP residents residing in congregated settings.

## 5 Discussion

This research describes how ongoing care deficits and the heightened threats asylum seekers and their children's health and wellbeing fell short of UN agency mandates, European CDC guidance and Ireland's HSE and Department of Justice and Equality recommendations. The health and human rights crisis of COVID-19 seems to have galvanized policy actors’ attention on the inhumane conditions of accommodation provided for asylum seekers and reinvigorated calls for improvements to the standard of care and replacement of the DP system.

The research contributes further evidence of the inability of IPAS, the HSE, DP centre staff and others to effectively adapt to an emerging health crisis during the initial waves of the COVID-19 pandemic. Failures of the State's ‘duty of care’ for people seeking international protection during COVID-19 are grounded in longstanding care deficits in DP, a system the Government assessed as ‘not fit for purpose’ (Government of Ireland, 2020: 7). At a most basic level, a state's provision of international protection implies ensuring people's safety. Yet research participants describe conditions in DP before, during and after COVID-19 as unsafe and re-traumatising.

### 5.1 Engendering an ethic of care in international protection

Ethics of care concepts frame ways to ensure a better standard of care that protects asylum seeker's health and wellbeing. Phases of care (Tronto, 1993) comprise ‘caring about, noticing the need to care in the first place; taking care of, assuming responsibility for care; care-giving, the actual work of care that needs to be done; and care-receiving, the response of that which is cared for to the care’ (ibid: 127). The findings of this demonstrates the DP system was unable to enact these standards. For example, the State outsources its responsibility for care to staff of DP centres who are not trained to practice trauma informed care and the way the system is structured subverts ways in which the ‘actual work of care’ can be delivered. Moreover, participants detailed their efforts to advocate for changes in conditions to IPAS with no effect thwarting the ability of care receivers to respond to the care that is provided to them .

A further phase of care (Tronto, 2013) considers states' role as providers of care and defines ‘caring with’ as a responsibility consistent with states’ ‘democratic commitments to justice, equality and freedom for all’ (Tronto, 2013: 23). The concept of a ‘caring democracy’ regards all people living in a state as equal recipients of care, assuaging tensions between autonomy and individual dependency which can undermine equality of access to health and social care services. It has been asserted that 'there is no caring justice, no justice open to recognizing the caring needs of all, including those who are today victims of neglect, harm or violence, outside a caring democracy' (Casalini, 2020: 68). These concepts support an emphasis on comprehensive care forasylum seekers in line with states’ political accountability for ‘national and international care responsibilities’ (O'Riordan, 2020: 4).

### 5.2 Policy implications

Several policy relevant recommendations emerge from these findings which could complement Ireland's aspiration towards an international protection system ‘grounded in a human rights approach’ (DCEDIY, 2021: 28). International protection should be enacted in ways that implement 'an approach to social justice capable of incorporating care as well as rights' (Barnes, 2006: 151). States must recognise that enactment of the value of justice for those seeking international protection exists within a moral context wherein care is the fundamental value (Held, 1995,^2006^). Ireland's international protection responsibilities must be practiced in ways that do not further harm applicants, do not exacerbate pre-existing or emerging health disparities nor re-traumatise asylum seekers.

Implementing enhanced standards of care to vulnerable people seeking a state's protection should not solely rely on system level reforms. The practice of care is within the remit of the people relating day-to-day with asylum seekers. Participants assert that they would like to relate to people who are more caring, who take greater care for and with them and urge people working within the mandate of the Ireland's international protection commitments practice care in the same way they would care for their own relatives. They expect public health standard are delivered in ways that safeguard them and not further exacerbate health disparities grounded in pre- and post-migration stressors and structural determinants.

The lack of care revealed in these findings, characterised by inflexibility to adapt public health recommendations issued in Irish, European and UN policy documents, must inform Ireland's Department of Justice and Department of Children, Equality, Disability, Integration and Youth to deliver their mandates in ways that care for and with asylum seekers and their children. Ensuring enhanced standards of care should be a key aspiration of efforts to replace Ireland's international protection accommodation system.

## 6 Strenghts and limitations

This paper responds to the need for qualitative research on how forced migrants endured the COVID-19 pandemic with an Irish study that considers structural determinants of health before, during and following the pandemic. It presents a Trace analysis of policy documents originating from the Irish Parliament's review of the COVID-19 response, considering the policy environment for delivering culturally competent health care and policy intentions to replace the existing international protection accommodation system and introduce an enhanced model of health care. The study's distinctive contribution to the field of migration health is the focus on how care ethics can inform international protection systems. While a discussion of how these findings compare to how other destination countries in the EU cared for asylum seekers during COVID-19 was not undertaken, Ireland has been regarded as ‘an interesting case as a small, open European country, which for the first-time experienced net inward migration in the past two decades’ (Mohan, 2021: 557).

The policy analysis is comprised of document review and did not engage policy actors, although some migrant rights organisations who were involved in policy processes reviewed endorsed the study aims and facilitated contact with participants. The empirical research comprises a small sample of 11 individuals and was carried out in 2022 some of time after events of the first wave of COVID-19 and lockdowns occurred raising the potential of recall bias amongst participants.

## 7 Conclusion

This research provides stark details on care failures during COVID-19 and beyond in Ireland's international protection accommodation services which are regarded as warehousing human beings. Public discourse and personal testimony reveal that care is not practiced with and for asylum seekers. and that systemic weaknesses in the two decades old ‘emergency’ system of DP render it a high risk congregated setting incapable of effectively implementing care standards robust enough to confront a public health crisis or avoid other structural determinants of health. The research adds evidence of the lack of trauma informed practices for asylum seekers coping with pre- and post-migration stress, a gap which pre-dates the onset of the pandemic and continues. The findings demonstrate that an inability to uphold an ethic of care results in failures the responsibility to protect asylum seekers’ health and wellbeing and respect their human dignity. which are relevant to efforts to reform how international protection responsibilities are enacted in Ireland and beyond.

## CRediT authorship contribution statement

**Felicity Daly:** Writing – review & editing, Writing – original draft, Formal analysis, Data curation, Conceptualization. **Jacqui O'Riordan:** Writing – review & editing, Methodology, Conceptualization.

## Declaration of competing interest

The authors declare that they have no known competing financial interests or personal relationships that could have appeared to influence the work reported in this paper.
